# Identifiability of Polychoric Models with Latent Elliptical Distributions

**DOI:** 10.1017/psy.2024.25

**Published:** 2025-01-03

**Authors:** Che Cheng, Hau-Hung Yang, Yung-Fong Hsu

**Affiliations:** Department of Psychology, National Taiwan University, Taiwan, R.O.C.

**Keywords:** elliptical distributions, identifiability constraints, just-identified models, polychoric correlations, polychoric models

## Abstract

The family of polychoric models (PM) categories ordinal data with latent multivariate normal variables. This modeling framework is commonly used to study the association between ordinal variables, often leading to a polychoric correlation model (PCM). Moreover, PM subsumes several well-known psychometric models, such as the structural equation modeling (SEM) with ordinal data. That said, the identifiability of PM has not been addressed in the literature. Meanwhile, in recent years researchers have suggested that the latent variables underlying PM could be generalized to the family of elliptical distributions, such as the multivariate logistic and *t* distributions. This article concerns the identifiability of PM and PCM with latent elliptical distributions, for which we show that PM is not identifiable and PCM is identifiable. In particular, we prove the identifiability of the polychoric *t* correlation model based on the copula representation. We then move on to find the set of identifiability constraints of PM through an “equivalence-classes approach of identifiability,” and demonstrate its use in two applications: one concerns the identifiability of PM on Likert scales and on comparative judgment, and the other concerns the identifiability of ordinal SEM and item factor analysis. Possible implications induced by these identifiability constraints are discussed.

Treating ordinal variables as interval or continuous variables might produce biased results (Olsson, 1979). The (family of) *polychoric models* (PM) deals with ordinal data by categorizing them with latent multivariate normal variables. PM mainly appears in two situations. First, PM is used to study the association between ordinal variables, often leading to a *polychoric correlation model* (PCM). PCM assumes that the latent variables underlying two ordinal variables follow the bivariate normal distribution with zero mean and a correlation matrix known as the *polychoric correlation* (Olsson, 1979). In particular, if both variables are binary, a polychoric correlation reduces to a tetrachoric correlation (Bonett & Price, 2005; Pearson, 1901).

Second, PM subsumes several commonly used psychometric models. In particular, PM is a general model of structural equation modeling with ordinal data (ordinal SEM; Muthén, 1984). Ordinal SEM models PM’s mean vector and covariance matrix as a function of some parameters. Several models are special cases of ordinal SEM, such as the graded response model (Samejima, 1968; Samejima, 1997) and the family of item factor analysis models (Wirth & Edwards, 2007).

Although PM is the basis of several models, its identifiability has not been addressed in the literature; only the identification of PCM (which, as mentioned above, is a special case of PM) has been proved (Almeida & Mouchart, 2003a). The importance of establishing the identifiability of a statistical model cannot be overstated. If a model is not identifiable, one may have two sets of parameters with the same probability distribution, even when the sample size approaches infinity. In this case, all estimators would not be consistent estimators.

To make the issue a bit more complicated, the normality assumption underlying PM has been challenged recently; researchers have suggested that the latent variables underlying PM could be generalized to the family of elliptical distributions, such as the multivariate logistic distribution and the multivariate *t* distribution (Jin & Yang-Wallentin, 2017; Kolbe et al., 2021; Roscino & Pollice, 2006). In light of this, in this article we explore these more general elliptical distributions. Two unsolved questions can be posited: (a) Are PM and/or PCM with latent elliptical distributions identifiable? (b) If any of them is not identifiable, can we find the minimal identifiability constraints? Practically, even if PCM is identifiable, it is not practical in some situations. For instance, when modeling the developmental changes of children, it is unreasonable to assume that all of the mean vectors are zero (McArdle et al., 2015; Muthén, 1984). Therefore, finding some other reasonable identifiability constraints is a task with practical significance.

This article aims at answering the above two questions and is organized as follows. Section 1 gives formal definitions of PM and PCM with elliptical distributions. We address the issue of (a) in Section 2. By generalizing Almeida and Mouchart’s (2003a) argument, we show that PCM with elliptical distributions is identifiable. In particular, we prove the identification of the polychoric *t* correlation model based on the copula representation. On the other hand, PM with elliptical distributions is not identifiable. We address the issue of (b) in Section 3. We show that one can find the identifiability constraints of PM through the equivalence-classes approach of Tsai (2000, 2003). This approach can also help determine the measurement scales of latent variables. The minimal identifiability constraints of PM on Likert scales and also on comparative judgment are demonstrated. Moreover, we prove a theorem stating the necessary and sufficient conditions for the identifiability of ordinal SEM and item factor analysis. Section 4 is devoted to the discussion of possible implications and applications induced by these identifiability constraints.

## 1 Definitions of PM and PCM with elliptical distributions

Suppose there are K ordinal-scale item scores, 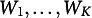 with support 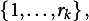 respectively. Let 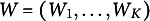 be the response vector. The family of polychoric models (PM) assumes that for an ordinal variable 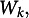 there is a corresponding vector of cut-offs (or thresholds) 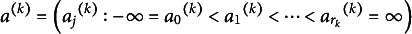 and a latent random variable 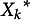 such that

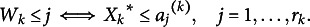



That is,
(1)

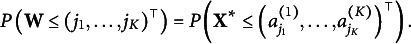

 where 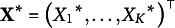 follows a distribution function 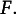 Thus, PM can be parametrized by

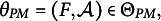

 where 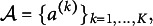 and 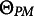 is the parameter space for this parametrization.

In the following, we review the concept of spherical and elliptical distributions with some examples.

Definition 1.1. (Fang et al., 1990)

- Let 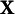 be a *p*-dimensional random vector. 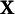 is said to have a spherical distribution if for any orthogonal transformation Γ, 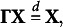 where 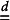 means “equal in distribution.”
- Let **Y** be an *n*-dimensional random vector. **Y** is said to have an elliptical distribution with parameters 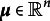 and 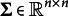 if

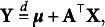

  where 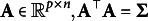 with 
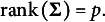

The following proposition characterizes the spherical and elliptical distributions.

Proposition 1.2. (Fang et al., 1990)

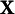
 is spherically distributed if and only if there is a scalar function 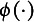 such that the characteristic function of 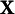 satisfies 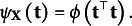 The function 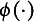 is called the characteristic generator of the spherical distribution.
- Under Definition 1.1(ii), The characteristic function of **Y** satisfies 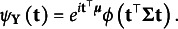 Therefore, an elliptical distribution can be parametrized by 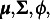 denoted 
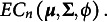

The parameters of (a family of) elliptical distributions are named below.

Definition 1.3.

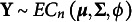
 , where 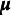 is the location vector, 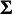 is the dispersion matrix or scatter matrix. If 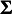 is positive definite, then 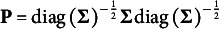 is called the *pseudo-correlation matrix*. If the first and second moments of **Y** exist, then 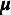 is called the *mean vector*, 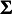 is called the *covariance matrix*, and 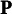 is called the *correlation matrix*.

Some commonly used elliptical distributions include the normal, logistic, uniform, and *t* distributions.

Example 1.4.

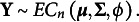
 Then **Y** follows

- a multivariate Normal distribution if 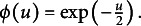 Also, we denote 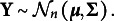

- a multivariate logistic distribution if 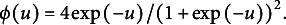

- a multivariate uniform distribution if 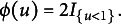

- a multivariate t distribution if 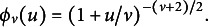 Also, we denote 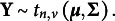 The characteristic function of a multivariate *t* distribution depends on the degrees of freedom parameter ν.

Definition 1.5.

- PM with a latent elliptical distribution characterized by 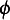 is 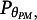 where 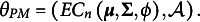

- PCM with a latent elliptical distribution characterized by 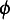 is 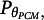 where 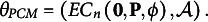

Unless specifically stated, in this article we study PCM and PM within the realm of elliptical distributions.

## 2 Identifiability of PCM with elliptical distributions

We first define some terms concerning model identifiability:

Definition 2.1.

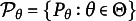
 is a parametric model. Let 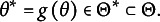 Then

- For any 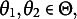 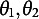 are empirically indistinguishable if 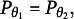 i.e., for any response w, 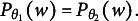

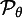
 is identifiable (or 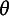 is identifiable) if 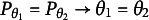 for all 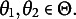

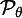
 is just-identified (or 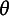 is just-identified) if 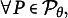 there is a unique 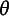 such that 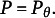

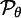
 is partially identifiable over 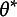 (or 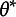 is partially identifiable) if 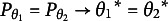 for all 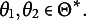

- For any 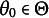 , the identified set of 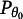 is 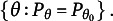

In ordinary language, two sets of parameters are empirically indistinguishable if they have the same probability structure for all possible outcomes. A model is identifiable if distinct sets of parameters correspond to distinct distribution functions; thereby, constructing consistent estimators is possible. Similarly, a model is partially identifiable over 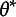 if distinct sets of 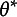 correspond to distinct distribution functions.

The following proposition states that if the model is parametrized by 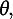 then the model is identifiable if and only if the model is just-identified. Therefore, if the model is parameterized by 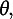 there is no need to distinguish between whether the model is just-identified or over-identified. The distinguishment between just-identified or over-identified is meaningful only when considering the restricted model of a parameterized model (see Section 3.1).

Proposition 2.2.

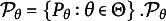
 is identifiable iff 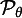 is just-identified.

Proof.

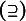
 The uniqueness of 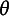 implies the identification of 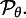

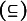
 The 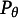 is parametrized by 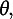 so for any 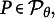 there is a 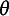 such that 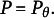 The uniqueness of 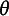 is guaranteed by the identification of 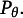

Note that parameter identification is a necessary condition for the existence of a consistent estimator. This proposition has been implicitly stated in some studies (such as Gu & Xu, 2019; Ouyang & Xu, 2022). San Martín and Quintana (2002) provided a formal formulation and a proof of the proposition (see below, where we also provide an alternative proof).

Proposition 2.3. (San Martín & Quintana, 2002) 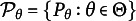 is a statistical model and 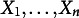 are independently, identically distributed random variables from 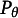 . Further, g is an invertible function of. If 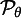 is not identifiable, then there is no consistent estimator of 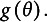 That is, the identifiability of the parameter is a necessary condition for the existence of a consistent estimator.

Proof. If the estimator 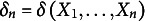 is a consistent estimator of 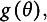 then 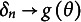 under 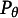 for any 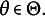 Given 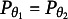 while 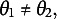 we have 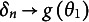 under 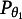 and 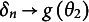 under 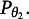 Because 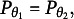 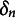 has the same distribution under 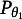 and 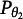 based on the inversion formula. Since a convergence sequence has a unique limit, 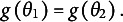 Because *g* is invertible, we have 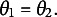 —a contradiction.

Identifiability is a property of a statistical model, sometimes defined as a property of parameters (Casella & Berger, 2002, p. 523). However, the statement “ 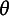 is identifiable” is ambiguous because it does not specify the statistical model in consideration. For example, Almeida and Mouchart (2003a) showed that PCM under the normality assumption is identifiable in PCM but not in PM. Indeed, the latter fact was derived from a proposition in Almeida and Mouchart (2003a).

Proposition 2.4. (Almeida and Mouchart, 2003a) For any monotonic increasing functions 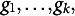 define 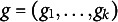 as a component-wise transformation. Then 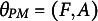 is empirically indistinguishable from 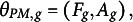 where 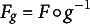 and 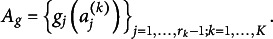

### 2.1 Olsson’s (1979) argument of identification of PCM

Olsson (1979) worked on the identification problem under PCM (with the normality assumption) by counting the difference between the number of parameters and the number of independent proportions. For convenience, we define the degrees of freedom, *df*, as

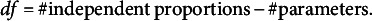



This definition is consistent with the common usage of degrees of freedom (Rodgers, 2019). Olsson (1979) argued that PCM is identified because 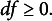 In particular, the model is *just-identified* if 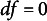 and *over-identified* if 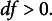 However, Proposition 2.4 implies that Olsson’s (1979) argument of identifiability is flawed, in that even for PCM under the normality assumption, 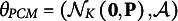 satisfying 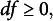 there could be a model 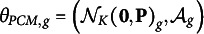 indistinguishable from it. Thus, merely observing 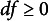 for a model does not guarantee that the model is identifiable. A similar statement (showing that 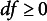 is a necessary but not sufficient condition for model identification) also can be found in the SEM literature (the *t* rule in Bollen, 1989).

In the literature, Almeida and Mouchart (2003a) provided the first rigorous proof of identifiability of PCM under the normality assumption. In the following, we generalize their proof to the elliptical distribution case. Specifically, using Proposition 2.2, we show that PCM is always just-identified under its model assumptions (see Theorem 2.5).^1^

### 2.2 Generalization of Almeida and Mouchart’s (2003a) proof

Motivated by Theorem 3.1 in Almeida and Mouchart (2003a), we now establish the conditions of identification under PCM with any latent continuous elliptical distributions.

Theorem 2.5. Under PCM with a latent elliptical distribution characterized by 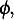 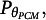 where 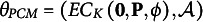 and 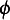 is known. If

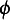
 defines a continuous distribution with strictly increasing univariate and bivariate CDFs on its support;
- the polychoric (pseudo-)correlation matrix 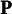 is positive definite,
- then PCM ( 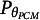 ) is just-identified.

Proof. It suffices to prove that if two pairs of parameters correspond to the same response probability vector, say, 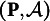 and 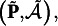 then 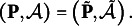 Our proof is composed of two parts:

- The cut-offs (or thresholds) are identifiable: Without loss of generality, consider the *j*th cut-off of item *k*, say, 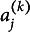 and 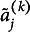 . Let the response vector 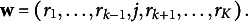 Then 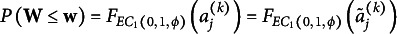 . Since the CDF is injective, 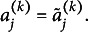 Because 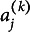 can be any cut-off in 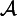 , we have 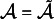 .
- The pseudo-correlation matrix is identifiable: Consider a response vector 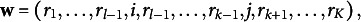 for which the *l*th response is *i*, and the *k*th response is *j*. We define 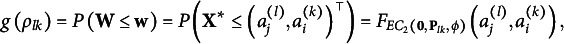 where 
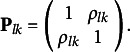
 Because 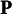 is positive definite, 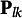 is also positive definite. So the Cholesky decomposition is unique: 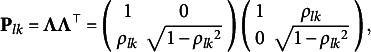 and 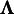 is invertible. We have 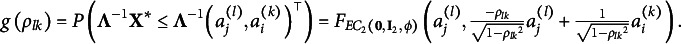

Since the bivariate CDF is monotone, 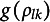 is strictly decreasing iff 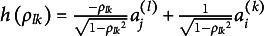 is strictly decreasing. Based on the calculus, we know that 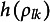 is strictly decreasing. Thus 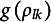 is strictly decreasing, and so 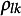 is identified. Because *i* and *j* can be any pair of items, we have 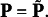

Based on Proposition 2.2, the model is just-identified because it is identifiable and also a parametrized model.

Corollary 2.6. The multivariate normal distribution, multivariate t distribution with known degrees-of-freedom, multivariate logistic distribution, and multivariate uniform distribution all satisfy the conditions in Theorem 2.5, so their corresponding 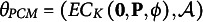 is identifiable. In particular, the multivariate normal case corresponds to Theorem 3.1 in Almeida and Mouchart (2003a).

Remark 2.7. The multivariate t distribution ( 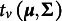 ) does not satisfy the conditions in Theorem 2.5, because the function 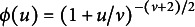 depends on the degrees-of-freedom parameter 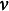 , which could be unknown.

As mentioned in Remark 2.7, Theorem **2.5** cannot cover the case of PCM with a latent multivariate *t* distribution (abbreviated as *polychoric t correlation model*). In the following, we provide a proof for this case based on the copula approach proposed by Almeida and Mouchart (2003b). For brevity, in the following paragraphs, we denote the CDF of a distribution simply by the symbol of that distribution. For example, we denote the CDF of the *t* distribution with degrees of freedom 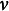 as 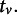 And the CDF of a multivariate *t* distribution 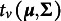 is abbreviated as 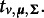


### 2.3 The copula approach to the identifiability of PCM

First, we introduce the concept of copula and define the copulas of elliptical distributions, under which the bivariate *t* copula will be a special case.

Definition 2.8. (Hofert, 2018) A copula is a multivariate CDF with standard uniform univariate margins, that is, the Unif(0,1) margins.

Definition 2.9. The elliptical copula characterized by 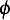 with *pseudo-correlation* 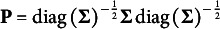 is defined as

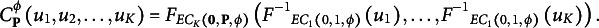

Definition 2.10. Let 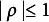 and 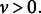 Then 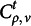 is a bivariate *t* copula if

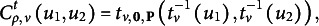

where 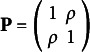 , 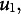 and 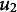 are within 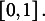

Remark 2.11. *The elliptical copula does not depend on the scale of the elliptical distribution. That is*,

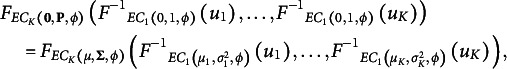

where 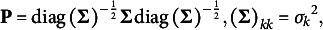 and 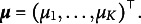 Therefore, to avoid over-parameterization, the parameters of elliptical copula do not contain 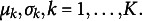

Almeida and Mouchart (2003b) found that PCM can be reparametrized by the copulas. Under this parametrization, the cut-offs are no longer the parameters to be considered. We formulate this property as a lemma.

Lemma 2.12. (Almeida & Mouchart, 2003b) Let 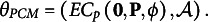 If 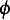 is a continuous distribution with strictly increasing univariate and bivariate CDFs on its support. Also, the k-th marginal is 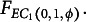 Let 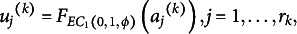 and 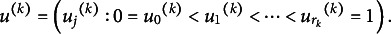 *Then PCM can be reparametrized by a* one-to-one correspondence function 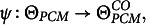 defined by 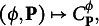 where

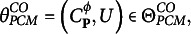

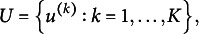
 and 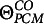 is the parameter space for this parametrization.

Proof. The proof can be derived from Equation (1):

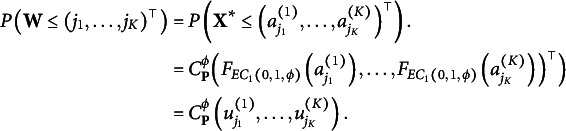

That is, two sets of parameters 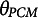 and 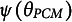 define the same CDF. Moreover, the assumption of 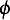 guarantees that 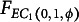 is invertible. It implies that the cut-offs can be uniquely determined through 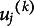 by 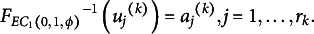 So the function 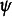 defines a one-to-one correspondence between two parameter spaces.

Using Lemma 2.12, in the following, we construct another proof of Theorem 2.5 based on the parametrization of copulas.

Proof of Theorem 2.5 based on copula parametrization. Based on condition (i) and Lemma 2.12, the identifiability of PCM under the copula parameterization 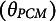 implies the identifiability of PCM 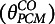 . We claim that if two pairs of copula parametrized parameters, 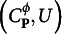 and 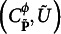 , correspond to the same response probability vector, then 
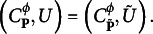
 This can be done by the following:

- The marginals are identifiable:

For any *k* and *j*, by definition, we have 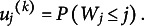 Based on the law of large numbers, 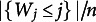 is a consistent estimator of 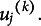 So based on Proposition 2.3, 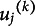 is identifiable.

- The pseudo-correlation matrix is identifiable:

Let 
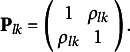
 Define 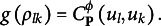 Similar to the proof of Theorem 2.5, we can show that 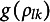 is strictly decreasing.

We note by passing that the identifiability of the pseudo-correlation matrix also leads to a property of elliptical copulas:

Corollary 2.13.

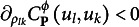
 for all 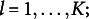 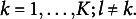

### 2.4 Identification of PCM with a latent multivariate t distribution

The following theorem establishes the identification of the polychoric *t* correlation model that Theorem 2.5 cannot cover. As aforementioned, the proof is based on the reparameterization of parameters by the copula.

Theorem 2.14. Let 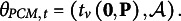 If the polychoric (pseudo-)correlation matrix 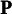 is positive definite, then the polychoric t correlation model ( 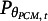 ) is just-identified.

Proof. If two pairs of parameters correspond to the same response probability vector, say, 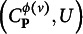 and 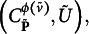 we claim that 
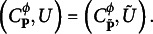
 Based on Lemma 2.12 and Corollary A.3, establishing 
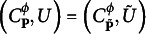
 suffices to complete the proof. Also, 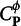 can be uniquely determined by all bivariate copulas implied by 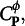 i.e., 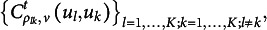 so establishing 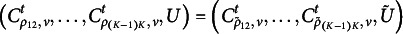 suffices to complete the proof.

Our proof is composed of two parts:

- The marginals are identifiable:

The proof is the same as the corresponding part of the “*Proof of Theorem 2.5 based on copula parametrization”* in the previous section.

- The bivariate copulas are identifiable:

Consider a response vector 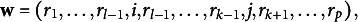 for which the *l*th response is *i*, and the *k*th response is *j*. We claim that both 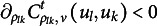 and 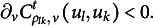 For 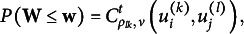 there is a unique copula 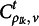 satisfying the equation. Thus 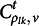 is identified, which completes this part of proof. The fact that 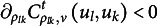 has been established by Corollary 2.13. It remains to show that 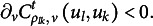 Let 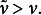 Without loss of generality, we may assume that both 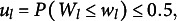 and 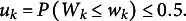 If 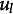 or 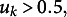 then the response can be reversely coded to achieve both 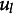 and 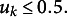 Because 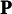 is positive definite, 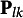 is also positive definite. Thus, the Cholesky decomposition 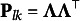 is unique and 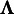 is invertible. Based on Lemma A.4 and the property of the multivariate *t* distribution, we have 
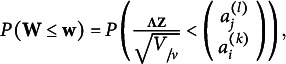
 where 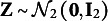 and 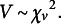 Therefore,

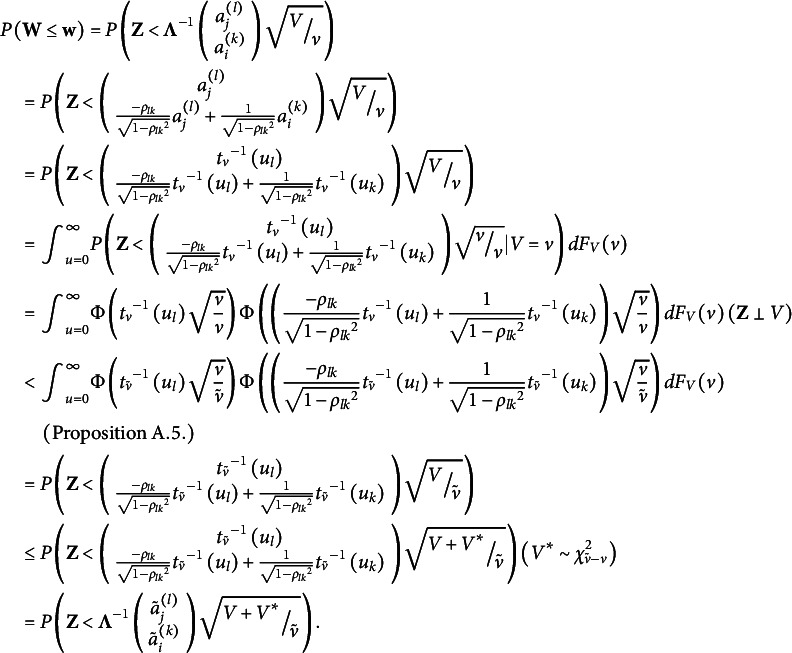



That is, 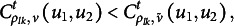 and so 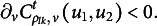 Because *l* and *k* are not specific, we have that 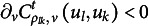 for any *l* and *k*.

We have proved that 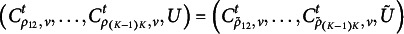 if two pairs of parameters correspond to the same response probability vector, and this leads to the identification of the polychoric *t* correlation model.

## 3 Identifiability and identifiability constraints of PM

In Section 2, we proved the identification of PCM. In comparison with PCM, the more general model PM is not identifiable, for which we now describe. We first present a proposition.

Proposition 3.1. For a polychoric model 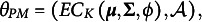 suppose the transformation 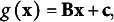 with 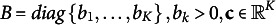 holds. Then 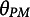 is empirically indistinguishable from 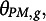 where 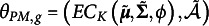 and

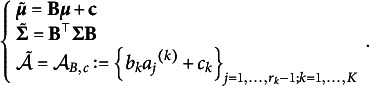

Proof. Let 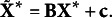 Then

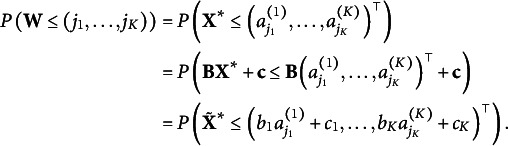

Corollary 3.2. *PM is not identifiable because* 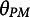 *and* 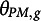 *are empirically indistinguishable, and both of them belong to* 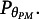

Thus, some workable identifiability constraints are needed to make PM identifiable. Indeed, the polychoric correlation model is constructed by putting some constraints on the location vector ***μ*** and the dispersion matrix **Σ** of PM. However, these identifiability constraints are not practical in some situations. For instance, when modeling the developmental changes of children, it is not well grounded to assume that the location vector ***μ*** is zero (McArdle et al., 2015; Muthén, 1984). Therefore, finding some other reasonable identifiability constraints is a task with practical significance.

### 3.1 The equivalence-classes approach of identifiability (ECAI)

An effective way to find the identifiability constraints was proposed by Tsai (2000, 2003) in the context of Thurstonian modeling of comparative judgment. Because this approach depends on the mathematical concept *of equivalence classes* (Dummit & Foote, 2004), we call it the *Equivalence-Classes Approach of Identifiability* (ECAI).

Traditionally, the identifiability of models needs to be proved case by case. ECAI provides a different route for derivation. Importantly, Tsai (2003) applied ECAI and proved that Case III and Case V of the Thurstonian models of comparative judgment are identifiable.

ECAI can help researchers to find a model’s identifiability constraints. Furthermore, it can be utilized to determine whether a (restricted) model is just-identified or over-identified. It utilizes the following two principles to construct the identifiability of the restricted model:

- For a non-identified general model, find the set of all parameters empirically indistinguishable from the true parameter, i.e., the identified set.
- After adding some constraints to the model, if there is at most one element in the predefined set, then the restricted model is identified, and the constraints are identifiability constraints. Moreover, if there is one and only one element in the predefined set, then the restricted model is just-identified; otherwise, the model is over-identified.

We can formulate ECAI as follows.

Proposition 3.3. Under Definition 2.1,

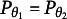
 defines an equivalence relation on 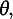 denoted by 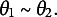

- The identified sets are the equivalence classes defined by the relation. Therefore, the identified set of 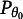 can be denoted as 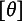 for all 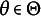 satisfying 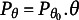 is called a representative of the equivalence class. That is, 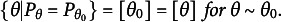

- The set of equivalence classes forms a partition of 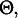 that is, for any 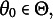 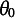 belongs to one and only one of the equivalence classes.

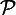
 is (just-)identifiable if and only if for each identified set 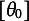 , there is a unique element, 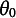 , in it, i.e., 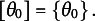 Namely, 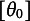 is a singleton.

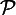
 is partially identifiable over 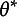 if and only if for each identified set 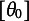 , there is a unique element in 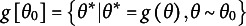 .

Proof. (i) is straightforward by the definition of the equality of functions. (iii) follows by Proposition 2 in 0.1 of Dummit and Foote (2004). (iv) and (v) can be proved by (iii), Proposition 2.2 and the definition of identifiability.

Proposition 3.3(iv) shows the sufficient and necessary condition for model identification. A statistical model might not be identifiable unless one adds some identifiability constraints to it. As mentioned above, Thurstonian models are identifiable with the Case III or Case V assumptions (Thurstone, 1927; Tsai, 2000). Here is another example. The confirmatory factor analysis model assumes that either the variance of the latent factor is any positive constant, or the loading of an indicator is any nonzero constant; otherwise, the model is not identifiable (Kline, 2023).

Definition 3.4. (Identifiability constraints) Under Definition 2.1, let 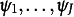 be functions of 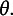 Then 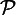 is identifiable with constraints 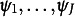 if the model with the parameter space under constraints, 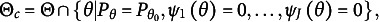 is identifiable. We call 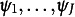 the identifiability constraints if 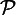 is identifiable with these constraints but not identifiable without these constraints.

Proposition 3.5. Let 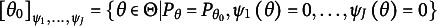 and 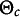 is not empty. The following three statements are equivalent:

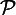
 is identifiable with constraints 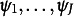

- For any 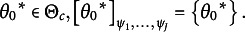

- For any 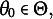 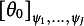 has at most one element.

Proof. (ii) can be easily derived from Proposition 3.3(iv). (iii) implies (ii) naturally. (ii) 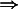 (iii) can be proved through contraposition. If there is a 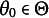 such that 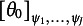 has more than one elements. Because 
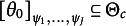
 , then (ii) is violated. Thus (ii) 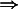 (iii).

The distinction between just-identified and over-identified (restricted) models can be recharacterized below using the concept of identifiability constraints.

Definition 3.6. Under Definitions 2.1 and 3.4,

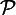
 is just-identified with constraints 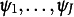 if for any 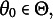 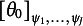 has a unique element. Moreover, 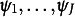 are called *minimal constraints* if removing any of it leads to non-identification.

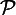
 is over-identified with constraints 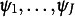 if there exists 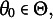 such that 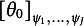 is empty.

We take the one-way ANOVA model (Rice, 2007) as an example to demonstrate the relation of non-identified, just-identified, and over-identified models. Let *K* be the number of groups. In this model, the parameters 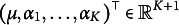 are not identifiable because both 
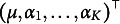
 and 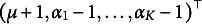 belong to 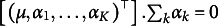 is an identifiability constraint because 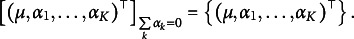 Also, “ 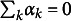 and 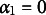 ” are identification constraints because a subset of the constraints ( 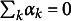 ) can identify the model. Moreover, 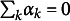 is a minimal constraint because for any 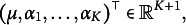 there exists a unique element 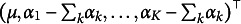 belonging to 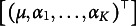 that removing 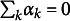 leads to non-identification. However, “ 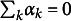 and 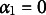 ” produce an over-identified model because there is a vector 
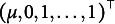
 such that 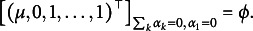 That is, there is no parameter which is empirically equivelent to 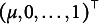 and simultaneously satisfies both 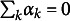 and 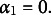


Another characterization of just-identification is the existence of a one-to-one correspondence between the identified parameters and the parameters with identification constraints (see Chang et al., 2017). It turns out this characterization is equivalent to the definition of just-identification:

Proposition 3.7. Under Definitions 2.1, 3.4, and 3.6., let the model with 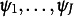 constraints be just-identified with the parameter space under constraints 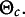 Then the model with 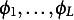 constraints is just-identified with the parameter space under constraints 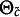 if and only if there is a one-to-one correspondence (of empirically indistinguishable models) between 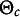 and 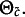

Proof.

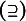
 Because the model with 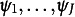 constraints is just-identified, for any 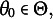 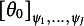 has a unique element. Then 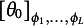 is also not empty based on the one-to-one correspondence. It indicates that the model with 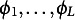 constraints is just-identified.

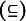
 Based on just-identification of the two models, both 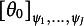 and 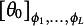 are singletons mapped by 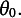 Thus, we can construct the correspondence between 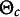 and 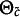 based on these mappings. This completes the proof.

The following theorem links ECAI to identifiability constraints.

Definition 3.8.

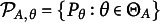
 and 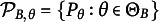 are two models where 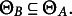 Then 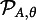 is a general model of 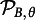 and 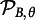 is a restricted model of 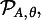 denoted by 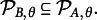

Theorem 3.9. Let 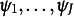 be functions of 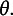 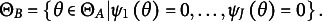 Thus 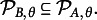 Then

- *If* 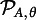 *is identifiable, then* 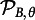 *is identifiable*.
- *If* 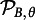 *is identifiable, then* 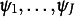 *are identifiability constraints of* 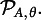 *In particular, If* 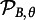 *is just-identified and removing any of* 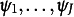 *leads to non-identification., then* 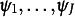 *are minimal constraints*.
- *(ECAI)* 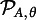 *is not identifiable*. 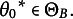 *Under* 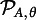 , if 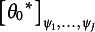 *is a singleton or empty, then* 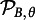 *is identifiable. Moreover*, 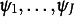 *are identifiability constraints of* 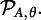 *Further, if* 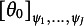 *is a singleton for any* 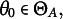 *then* 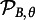 *is just-identified. Otherwise* 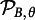 *is over-identified*.

Proof. The results are immediate consequences of Proposition **3.3** (iv) and Definition 3.6.

Another related issue is that the equivalent class of a non-identifiable model consists of all permissible transformations of a model, which can determine the scale of measurement (Luce et al., 1990, Chapter 20; Stevens, 1946). The scale of a measurement can be identified as follows.

Definition 3.10. Let 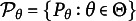 be a model and X be a random variable 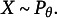 Define 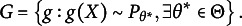

- *X* is a nominal scale if 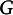 consists of all injective functions.
- *X* is an ordinal scale if 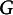 consists of all monotone increasing functions.
- *X* is an interval scale if 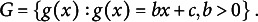

- *X* is a ratio scale if 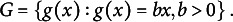

- *X* is an absolute scale if 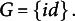

### 3.2 Identified sets of PM

Theorem 3.11 finds the equivalent class for PM. The theorem states that all empirically equivalent PMs can be linearly transformed into each other. So, the equivalent class of PM is the set of all possible linearly transformed PMs of it, and the latent vectors corresponding to PM are interval scales.

Theorem 3.11. *Given the conditions in Theorem 2.5, consider PM* 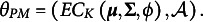 *Also*,
(2)

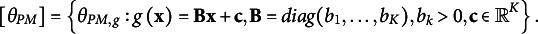

Then all entries of 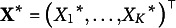 are interval scales, and the equivalent class of PM consists of models 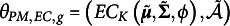 *satisfying*


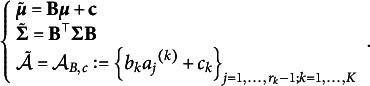

Proof.

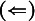
 is straightforward by Proposition 3.1.

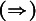
 Consider two PMs, 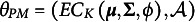 and 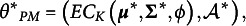 where 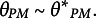 We claim that

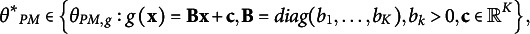

that is, there is a function 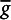 such that 
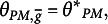
 where 
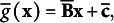
 Define 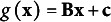 where 
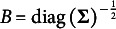
 and 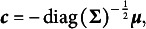 and 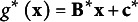 where 
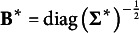
 and 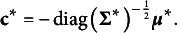 Based on Proposition 3.1, we have 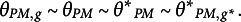 Both 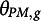 and 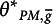 are PCM. Because of the identification of PCM (i.e., Theorem 2.5 and Theorem 2.14), there cannot be two different PCMs that are empirically indistinguishable. Therefore 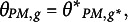 and so 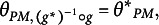 i.e., 
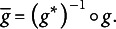

By applying Proposition 3.7, Theorem **3.9**, and Theorem 3.11, we show in the following that the constraints of 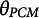 are minimal constraints. We then construct the criteria for the minimal constraints of the identifiability constraint in PM.

Corollary 3.12. For PM the constraints that 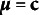 and 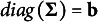 (where 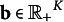 with 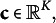 ) are the minimal constraints. In particular, for PCM, the constraints that 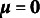 and 
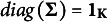
 are the minimal constraints.

Proof. Equation ( 2) shows that 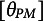 will not be a singleton when any of the two constraints 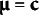 and 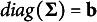 is removed. Therefore 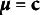 and 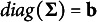 consist of the minimal constraints. The particular scenario concerning the minimal constraints of PCM is also supported by the application of Theorem 3.9(ii).

Corollary 3.13. Under PM, let 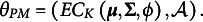 The following three statements are equivalent:

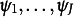
 are the minimal constraints.
- There is a bijection from 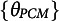 to 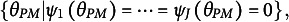 and removing any of 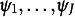 leads to non-identification.

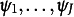
 are identifiable constraints, there is a surjection (of empirically indistinguishable models) from 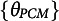 to 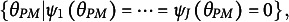 and removing any of 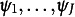 leads to non-identification.

Proof.

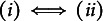
 By Proposition 3.7 and Corollary 3.12, 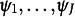 are the minimal constraints if and only if there is an injection from 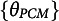 to 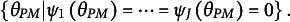

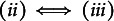
 The identification can guarantee the injection. The reverse is also true.

In the following, we demonstrate two applications of the ECAI approach along with our theorems concerning the identified sets of PM. First, we will use ECAI to find the identifiability constraints of PM on Likert scale (LS) and on comparative judgment (CJ) items. Second, using the ordinal SEM and item factor analysis as an example, we will illustrate the use of ECAI in establishing the identifiability of (restricted) models of PM.

### 3.3 Application 1: Identifiability constraints of PM on Likert scales and on comparative judgment

As mentioned earlier, PM subsumes several commonly used psychometric models in analyzing LS and CJ items. For LS, the graded response model (Samejima, 1968; 1997) and item factor analysis models (Wirth & Edwards, 2007) are restricted PMs. For CJ, Thurstonian models (Thurstone, 1927) and their advances (Bockenholt & Tsai, 2001; Brown & Maydeu-Olivares, 2013) are also restricted models of PM. The following theorem shows some identifiability constraints of PM on LS and CJ items. For convenience, CJ with *r*-point ordinal preference responses is called *r-point CJ* (Agresti, 1992; Brown & Maydeu-Olivares, 2018), whereas LS with *r*-point ordinal responses is called *r-point LS*.

Theorem 3.14.

- Consider K items in a Q-point LS (Q > 2). If the first cut-offs of items are set to zero, and the final cut-offs are set to one ( 
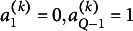
 *), then these constraints are minimal, and the PM is just-identified. Moreover, all entries of* 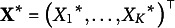 *are absolute scales. We call the above constraints the “global scale constraints (GSC).”*
- *Consider K items in a (2H)-point LS/CJ (H ≥ 1). If the middle cut-offs of LS/CJ items are set to zero (* 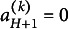 *), then* 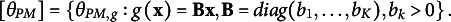 *So all entries of* 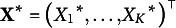 *are ratio scales. Moreover, if* 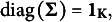 *namely,* 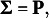 *then these constraints are minimal and the PM is just-identified. We call* 
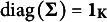
 the “unit variance constraints (UVC)” because it assumes that the variances of scales in X_1_* to X_k_* are one.
- *Consider K items in a (2H + 1)-point LS/CJ items (H ≥ 1). If the middle cut-offs of LS/CJ items are symmetric around 0 (* 
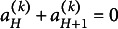
 *), then* 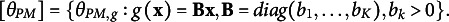 So all entries of 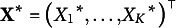 *are ratio scales. Moreover, if* 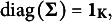 *namely*, 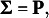 *then these constraints are minimal and the PM is just-identified.*
- *Consider K items in a Q-point LS/CJ (Q > 2). If the cut-offs of extreme preferences are set to be − 1 and 1 (i.e.*, 
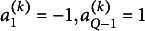
 *), then these constraints are minimal and the PM is just-identified. Moreover, all entries of* 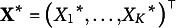 *are absolute scales. We call the above constraints the “extremity constraints (EC).”*

Proof. Because 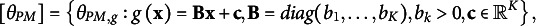 we only need to consider whether 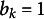 and 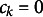 for all *k*. If both hold, then any PM-based model with these constraints is identifiable. Moreover, by Corollary 3.13, if there is a surjection from PCM to PM with the aforementioned constraints, then the PM is just-identified.

- For any k, 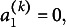 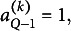 so

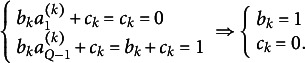

Therefore 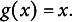 For any PCM, we can construct a surjection by rescaling 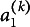 to 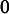 and 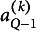 to 1 for all items. Therefore, the model is just-identified. These constraints are minimal because they are linearly independent.

- For any k, 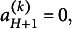 and 
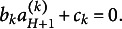
 So 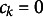 for all *k*. We now prove the identification under the UVC. Let 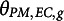 be empirically indistinguishable from 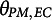 for some 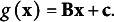 Because 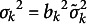 and 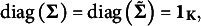 we have 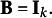 For any PCM, we can construct a surjection by shifting 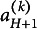 to 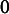 for all items. Therefore, the model is just-identified. These constraints are also minimal because they are linearly independent. (iii) can be proved in a similar way.

(iv) For any k, 
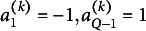
 so

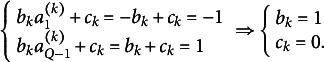

Therefore 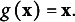 For any PCM, we can construct a surjection by rescaling 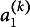 to −1 and 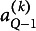 to 1 for all items. Therefore, the model is just-identified.

Theorem 3.14(i) and (iv) show that PM is identifiable with GSC or EC for LS items. Indeed, GSC or EC would be appropriate for LS with different context labels. For instance, Casper et al. (2020) enumerated several types of labels, such as agreement, similarity, and frequency. Within this context, EC might be better than GSC for the agreement anchor because agreement and disagreement are two extreme attitudes. For example, for a 7-point LS from 1 (strongly disagree) to 7 (strongly agree), we could set the bound of responding “strongly disagree” to −1 and “strongly agree” to 1. In contrast, for the similarity and frequency anchor, GSC might be better than EC, because GSC is better at representing the context of similarity or frequency. For example, for a 7-point LS from 1 (not at all like me) to 7 (extremely like me), we could set the cut-offs of “not at all like me” to 0 and “extremely like me” to 1.

Under GSC, because the zero and unit are prespecified, the scale is an absolute scale, i.e., a scale with an absolute zero and absolute unit (Zwislocki & Goodman, 1980). The absolute scale is a type of measurement scale that extends Stevens’ (1946) classification (Luce et al., 1990). An absolute scale might be helpful in practice because all calculations are permissible for the absolute scale.

Moreover, Theorem 3.14(ii) and (iii) show that PM is identifiable with UVC or EC for CJ items. Under the Thurstonian modeling framework, 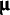 is the differences of the means of latent processes. In order to overcome the identifiability problem, researchers have made assumptions such as Case III or Case V to identify the model parameters (Thurstone, 1927; Tsai, 2000). Here we demonstrate that other constraints, such as UVC and EC, can help researchers to identify 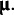 (Note that the latent scales under PCM and UVC share a common unit but have different origins. The relation between these two is similar to a standardized scale 
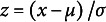
 and a scale normalized by the standard deviation: 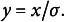 ).

We mentioned earlier that Case V implies the covariance matrix underlying CJ items 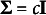 for some c, where 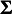 is the covariance matrix of latent differences (Thurstone, 1927; Tsai, 2000). Let the variance of the discriminal process be 1/2, then 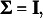 indicating that UVC ( 
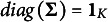
 ) is a general model of Case V. The identification of UVC implies that the independence assumption in Case V is unnecessary for identifying the covariance matrix underlying CJ items. Assuming UVC rather than Case V can avoid the risk of making wrong uncorrelated assumptions. In practice, UVC can be applied to multidimensional scaling (MDS). Recall that MDS utilizes 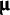 to infer multidimensional relative positions among different alternatives, for which Case V is typically assumed (Torgerson, 1952). UVC can identify 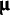 without assuming that the latent variables are uncorrelated.

### 3.4 Application 2: Identifiability of restricted models of PM

PM subsumes several commonly used psychometric models. We can use this hierarchy to prove the identifiability of some commonly used psychometric models. We first prove a lemma.

Lemma 3.15. Let 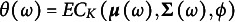 be an elliptical distribution parametrized by a real vector 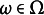 , then the empirically indistinguishable relation defines the family of equivalence classes 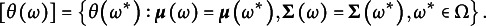 Consequently, the equivalence class of the PM with parameter 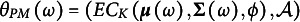 is
(3)

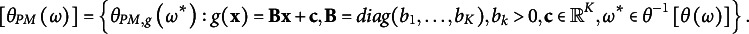

Proof. Based on Theorem 3.11, 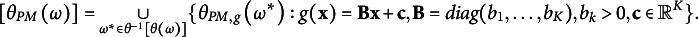 The union of equivalence relations satisfies reflexivity and symmetry natually. Also, the equivalence of probability structure satisfies transitivity. Thus, the union froms another equivalence class.

Based on the above lemma, we can easily derive that If 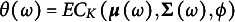 is just-identified/identifiable, then PM with parameter 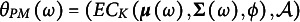 is an interval scale. An application is stated in the following theorem, which presents necessary and sufficient conditions for the identifiability of the ordinal SEM. Notably, the item factor analysis (FA) model, also named as the confirmatory multidimensional item response theory (IRT), can be granted as a special case of ordinal SEM (Asparouhov & Muthén, 2020; Reckase, 2009; SAS Institute Inc., 2024; Takane & de Leeuw, 1987; Wirth & Edwards, 2007).

Corollary 3.16. An SEM model, 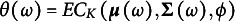 , is identifiable if and only if the corresponding ordinal SEM model is an interval scale.

Proof.

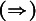
 Based on the identification of the SEM model, there is only one element, 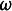 , in 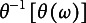 . Thus Equation (3) reduces to 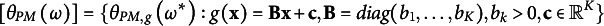 , which is the definition of the interval scale. Reversely, if the ordinal SEM model is not an interval scale, then 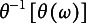 has more than one elelments. Thus, the SEM model is not identifiable.

Theorem 3.17. (Sufficient and necessary condition for (just-)identifiability of ordinal SEM) The ordinal SEM model, 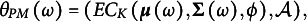 with constraints 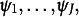 is identifiable if and only if the SEM model is identifiable and 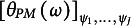 admits both 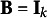 and 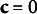 in Equation (3). Moreover, it is just-identifiable if and only if the SEM model is just-identifiable and for any 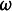 such that 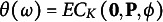 there is one and only one corresponding 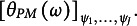 In addition, 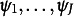 are minimal constraints if removing any of 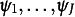 leads to non-identification.

Proof.

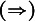
 Using an argument similar to that of Corollary 3.16, the ordinal SEM model with constraints admits a partition finer than that of an interval scale, and so the SEM model is identifiable. Moreover, we have

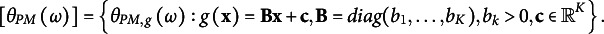

Now that the ordinal SEM model is identifiable. It implies 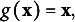 which is equivalent to 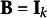 and 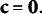

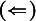
 Because the SEM model is identifiable, and 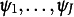 admits both 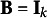 and 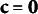 , the equivalence class 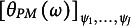 will not have more than one element. Therefore, the ordinal SEM model is identified.

The remaining parts can be easily proved through Corollary *3.13*.

Corollary 3.18. (Sufficient and necessary condition for (just-)identifiability of item factor analysis) An item factor analysis model (setting all of the error variances to be one) is identifiable/just-identifiable if and only if the corresponding factor analysis model (through correlation matrix) is identifiable/just-identifiable.

Proof. Consider an *m*-dimensional item factor analysis with discriminant parameter vectors 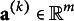 and threshold vectors 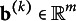 for item *k*, *k* = 1,…,*K*. Based on the formulas (6) and (13) in Takane and de Leeuw (1987), the model can be expressed as
(4)

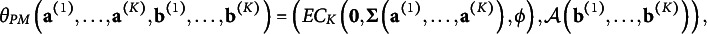

where 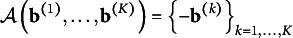 and 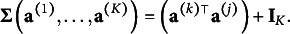 We now check the conditions in Theorem 3.17. Because there is a one–one correspondence (through standardization and its inverse transformation) between 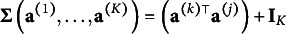 and the FA model through correlation matrix, 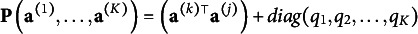 , we can reparametrize the model as a PCM. Thus, it is identifiable/just-identifiable when the corresponding factor analysis model (through correlation matrix) is identifiable/just-identifiable.

Remark 3.19. Based on the formulas (6) and (13) in Takane and de Leeuw (1987), the model can be parameterized as an IRT or as an FA. The IRT parametrization corresponds to Equation ( 4), and the FA parametrization corresponds to the following equation

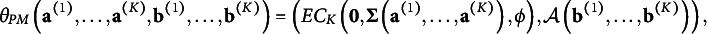

where 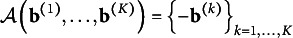 and 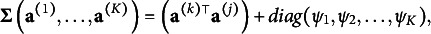 where 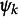 is the error variance corresponding to item k. Note that the FA parametrization also corresponds to the theta parametrization in MPLUS (Asparouhov & Muthén, 2020). Because 
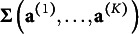
 corresponds to more than one 
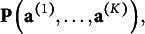
 the FA parametrization is not identifiable unless one sets 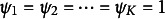 (Takane & de Leeuw, 1987).

## 4 General Discussion

Two unsolved questions concerning the identifiability of PM or PCM have been raised: (a) Are PCM and/or PM with latent elliptical distributions identifiable? (b) If any of them is not identifiable, can we find the minimal identifiability constraints? For (a), we proved the just-identifiability of PCM and non-identifiability of PM by generalizing Almeida and Mouchart’s (2003a) argument. In particular, we proved the just-identification of the polychoric *t* correlation model based on the copula representation. For (b), we found the sets of identifiability constraints of PM using ECAI of Tsai (2000, 2003). Our results showed that PM of LS with GSC or EC is just-identified, and that PM of CJ with UVC or EC is just-identified. Moreover, all of GSC, EC, and UVC are minimal constraints for identification. We also showed that for CJ, the latent differences underlying the Thurstonian models are ratio scales.

Identifiability is the premise of constructing consistent estimators. While researchers have advanced in proving the identifiability of several commonly used psychometric models (Fariña et al., 2019; Gu & Xu, 2019; Ouyang & Xu, 2022), the issue of non-identified models has not been fully addressed. ECAI provides an alternative by focusing on model hierarchy and can help researchers find identifiability constraints of non-identified general models. In particular, we showed in Theorem **3.9** (i) that if the general model is identifiable, then the restricted model is identifiable. This theorem justifies the common practice that if the most general model among a set of model hierarchies is identifiable, then there is no need to prove the restricted models in the model hierarchy. For example, if a confirmatory factor analysis model is identifiable, then the model with more constraints is also identifiable.

The scales under PM with GSC or EC have more merits than PCM. Linking the results from two LS attitude tests might be more manageable. If the labels of the extremities of the anchors in the two LS tests are the same, then the two constructed polychoric models will have the same unit. We may assume that people have a consensus on understanding what the anchors mean. For example, the corresponding PM may have the same unit if two tests with the same LS anchor range from 1 (strongly disagree) to 7 (strongly agree). Alternatively, if two LSs have different anchors or different numbers of points, then these two scales might have different scale origins and units. Future studies should investigate how to equate two LS scales with different anchors or different numbers of points.

Traditionally, most researchers adopt PCM rather than PM to model LS. For researchers interested in modeling changes in longitudinal data, however, PCM does not meet the need to identify mean differences among different ages. Instead, researchers usually adopted PM with some scalar invariance assumptions to identify the mean differences (McArdle et al., 2015; Muthén, 1984). If longitudinal studies can adopt GSC, then the mean differences among different ages can be identified without further assumptions.

Identifiability has been an issue for Thurstonian models. Theorem 3.14(iv) provides a novel approach to the identifiability problem. If one adopts CJ items with more than two points, then PM is identifiable with EC. The intuition behind EC is that we define −1 and 1 as the points when a participant shows the strongest preference for one over another. Because EC can only apply to CJ with more than two points, we suggest researchers adopt CJ with more than two points for identifiability purposes. Similar to the LS case, if two CJ items have different numbers of points, they have two different units. Future studies should also investigate how to equate these two scales constructed by CJ items with different numbers of points.

Moreover, we have proved the necessary and sufficient conditions for the ordinal SEM model to be just-identifiable/identifiable. Specifically, we demonstrated that when the FA based on the correlation matrix is identifiable, the item factor analysis model within an IRT parametrization is also identifiable. However, the model within the FA parametrization remains unidentifiable unless all error variances are fixed at one. This result is consistent with the established literature about item factor analysis (Takane & de Leeuw, 1987; Wirth & Edwards, 2007). These theorems might help investigators to determine the identification of more advanced ordinal SEM models. Also, we note that these theorems release the normality assumption of ordinal SEM, providing more flexibility for statistical modeling.

We have focused on the identifiability issue of PCM and PM with latent elliptical distributions. Distributions not belonging to the family of elliptical distributions, such as the skewed normal distribution (Jin & Yang-Wallentin, 2017), need further investigation.

**Figure 1:**
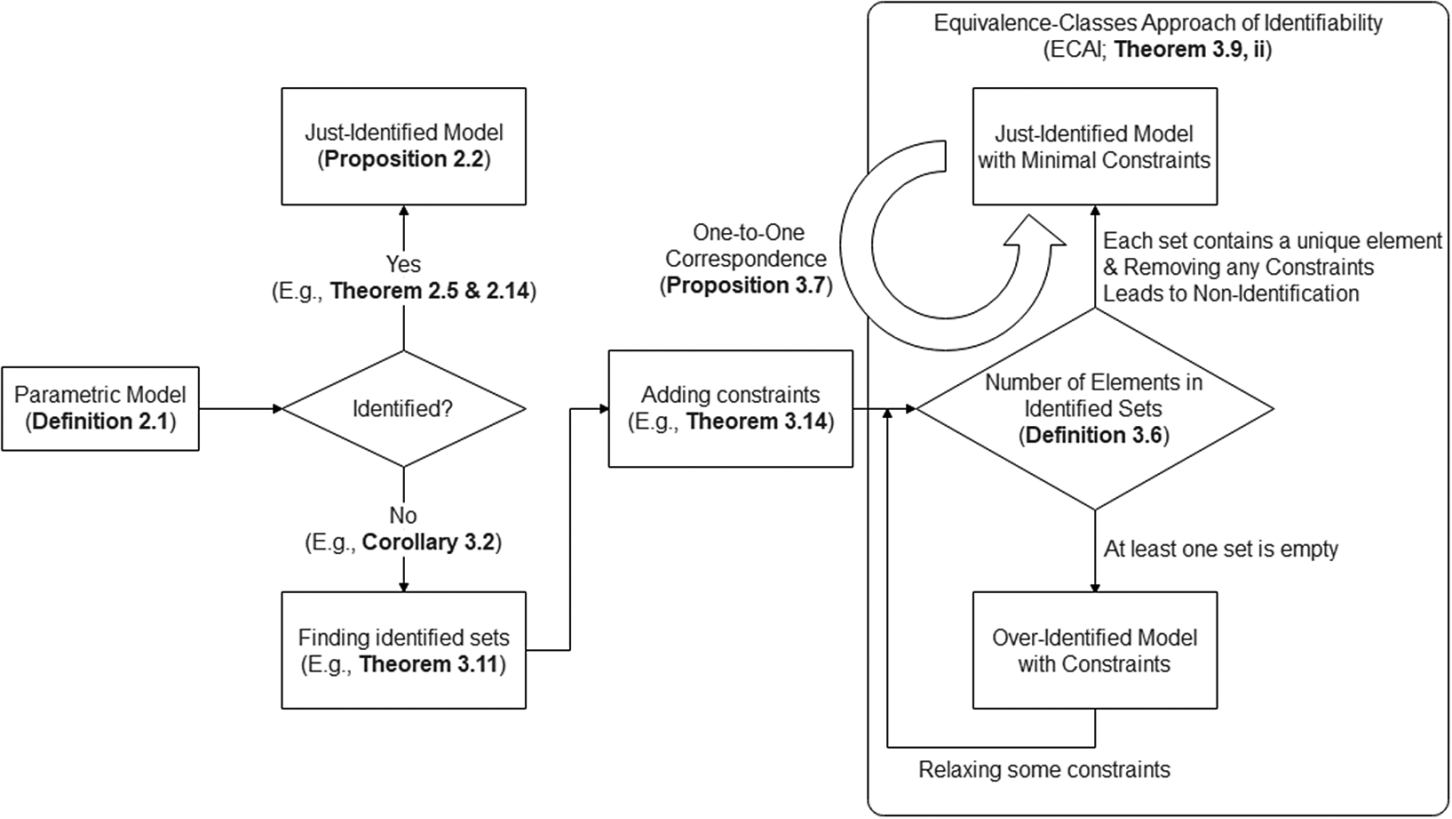
A flowchart for establishing model identifiability and finding identifiability constraints. *Note:* In the flowchart, each process is annotated with the corresponding theorem(s) and example(s) in this article.

We mention that the approach we proposed in this article is not confined to the identifiability issue of PM and/or PCM models. In Figure 1, we provide a flowchart for establishing model identifiability and finding identifiability constraints, in which each process is annotated with the corresponding theorem(s) and example(s) in this article. The steps of this principled approach are as follows. (1) Select a parametric model. If it proves to be identifiable, then it is deemed just-identified. If, however, the model is not identifiable, we search for identifiability constraints through ECAI. (2) Upon applying some constraints, if each identified set contains at most one element, then the model is identified. If, on the other hand, at least one set is empty, then the model is over-identified, which implies a probable need to relax some constraints. (3) If each set contains a unique element and removing any constraint makes the model non-identifiable, then the model is just identified and these constraints are minimal constraints.

In this article, the concept of identifiability discussed is global identifiability. There may be some weaker conditions that might be useful. First, some commonly used latent variable models struggle to ascertain whether they are globally identifiable. In such cases, researchers can verify whether the model possesses the characteristics of local identifiability (see Bekker et al., 1994; Skrondal & Rabe-Hesketh, 2004). Second, we may conceive a weaker concept of identifiability from lower-order margins. In this study, the polychoric correlation models show “identifiability from bivariate distributions.” In such a scenario, it may be possible to find consistent estimators of the parameters by only assuming bivariate distributions (without assumptions about higher-order joint distributions).^2^ These estimators, which coincide with the limited-information estimator of the elliptical cases, may lead to robust estimators. These discussions potentially open a new direction for future research.

## Figures and Tables

**Figure A1:**
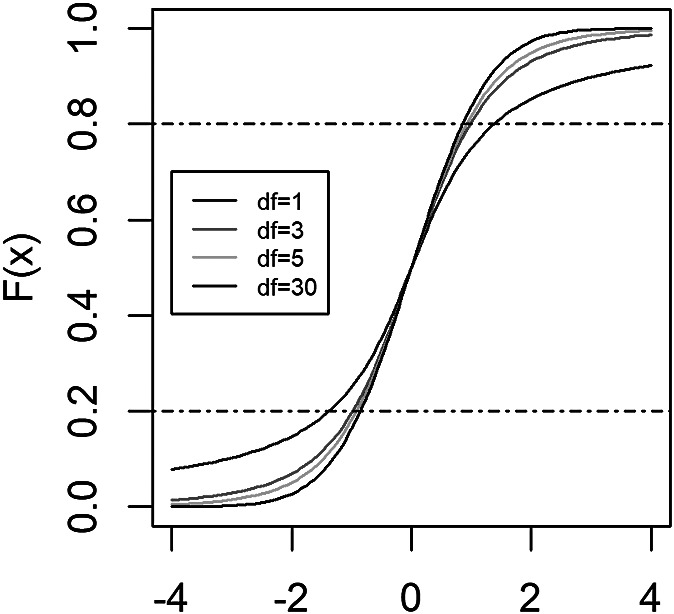
The CDF of the *t* distribution with different degrees of freedom. *Note:* Fixing the target probability 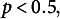 then the quantile is a strictly increasing function of ν; if 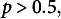 then the quantile is a strictly decreasing function of ν.

## Data Availability

Data sharing is not applicable to this article as no real datasets were collected in the study.

## Footnotes

1
At first glance, the statement of just-identifiability of PCM seems somewhat counter-intuitive, especially it is contradictory to the statement that  However, we only state that PCM is just-identified under its model assumptions, but not under a more general framework. For instance, consider a set of data consisting of *K* ordinal-scale items, and each item has  possible categories with probability  Then we can treat PCM as a model nested in this framework. In such a case, PCM may not be just-identified (see Almeida and Mouchart, 2003a).

2
We thank one of the reviewers for pointing this out.

## Appendix

The statements shown here are used in the proof of Theorem 2.14 for the identification of PCM with a latent multivariate *t* distribution. Lemma A.1.
*Suppose that X and Y are independent random variables with*   *for*  *Then*

Lemma A.2 is used to establish the monotonicity of the CDF of the product of a jointly distributed random variable. Lemma A.2.
*If X and Y are two independent, continuous random variables and*  *then Z is a continuous random variable. Moreover, if X is supported on the positive values and Y is supported on the whole real line, then Z is supported on the whole real line, and the CDF of Z is strictly increasing and injective on the real line*.
Proof. Suppose that there exists  such that  Because  we have  almost everywhere. However,  and  are nonzero almost everywhere, so  cannot exist. Thus *Z* is supported on the whole real line. Now because for any *b* > *a*, where *ϵ* is the infimum of within [*a*, *b*]. Thus the distribution of *Z* is strictly increasing, and so the function is injective on the real line.
Corollary A.3.
*A t distribution is supported on the whole real line, and the CDF is strictly increasing and injective on the real line.*
Lemma A.4.
*Let*  *be the CDF of a t distribution with degrees of freedom*  *Then for any*

*Therefore*,  *and*  *have the same sign. More generally, let*  *be the CDF of a multivariate t distribution*  *, and*  *If*  *then*

Proof. By the definition of *t* distribution, if  then  for some  and  Without loss of generality, for any

Likewise, for any  The multivariate case can be proved using a similar method.

Proposition A.5 shows that the quantile of the *t* distribution is an implicit function of the degrees of freedom. Figure 1 demonstrates this property. Proposition A.5.
*Let q be the u-quantile of the t distribution, i.e.*,  *Given u, if*  *then*  *is strictly increasing and invertible on*  *if*  *then*  *is strictly decreasing and invertible on*  *In other words, for any*

Proof. Fix  and let  and  be the *u* quantile of *t* distributions with degrees of freedom  and  , respectively. The uniqueness of the quantiles is guaranteed by Corollary A.3. Also, based on the property of *t* distributions,  Then, based on Lemma A.4, for any  By Corollary A.3,  therefore,  is strictly increasing. Similarly, for   is strictly decreasing. Also, for any fixed values of *u* and  there is a value  such that  Thus  is invertible on both  and
